# The Features of Phase Stability of GaN and AlN Films at Nanolevel

**DOI:** 10.3390/nano11010008

**Published:** 2020-12-23

**Authors:** Ilya V. Chepkasov, Sergey V. Erohin, Pavel B. Sorokin

**Affiliations:** 1 Inorganic Nanomaterials laboratory, National University of Science and Technology “MISIS”, Leninsky Prospect 4, 119049 Moscow, Russia; ilyachepkasov@gmail.com (I.V.C.); sverohin@tisnum.ru (S.V.E.); 2 Center for Energy Science and Technology, Skolkovo Institute of Science and Technology, 121025 Moscow, Russia; 3 Department of Structural Research, Technological Institute for Superhard and Novel Carbon Materials, Troitsk, 108840 Moscow, Russia

**Keywords:** GaN, AlN, wurtzite, body-centered-tetragonal phase

## Abstract

Recently, two-dimensional gallium and aluminum nitrides have triggered a vast interest in their tunable optical and electronic properties. Continuation of this research requires a detailed understanding of their atomic structure. Here, by using first-principles calculations we reported a systematic study of phase stability of 2D-GaN and 2D-AlN. We showed that the films undergo a phase transition from a graphene-like to a wurtzite structure with a thickness increase, whereas the early reported body-centered-tetragonal phase requires specific conditions for stabilization. Additionally, we studied how the functionalization of the surface can modify the film structure as exemplified by hydrogenation.

## 1. Introduction

The detailed study of graphene, discovered in 2004 [1], led to the emergence of a new materials family consisting of hundreds of species that formally have a two-dimensional structure. The decrease of thickness down to the atomic level excludes the bulk contribution and keeps only the surfaces (and edges) that give access to fascinating effects. Such unique possibilities have inspired the scientific community to search for new 2D films and synthesis of ultra-thin materials which do not have an initially layered structure as well as to investigate the influence of size effects on the atomic structure. The usual problem in the study of such materials is understanding of the film structure at the nanoscale level. The film thickness adds a new valuable term to the whole energy balance associated with the surface effects which even can switch the lattice organization throughout. Such an effect can be well illustrated by the graphitization of 2D diamond films and their reverse formation from graphene by reference atoms adsorption [2,3,4] or by splitting of ultrathin films with polar surface [5,6,7].

The direct impact of surface conditions to thermodynamics is especially important for the recently produced 2D gallium and aluminum nitrides [8,9,10,11]. The wide applications of their bulk counterparts stimulated a natural interest to the properties of 2D films. For example, the amplitude, onset energy of the absorption spectrum and the absorbance can be controlled by changing the number of 2D GaN and AlN layers [12]. Due to the quantum confinement effect, atomically thin III-nitride semiconductors regarded as 2D materials display an ultrawide bandgap [8,13], which can be used in next-generation optoelectronic devices [14,15]. Of particular interest are AlN/GaN heterostructures, where the ultrathin GaN representing some sort of quantum well confined between AlN barrier layers. In this case, the bandgap can achieve up to 5 eV or more [16,17]. Such tuning of the band structure can broaden the application area of GaN, AlN films, and offers unique opportunities in ultra-fast tunneling technology and inter-sub-band devices [18]. According to Wang et al. [19] AlN monolayers are suitable for detecting H_2_, CO, CO_2_, NO, and O_2_ via charge transfer mechanism. What seems especially intriguing is the specific structural film transformation at the nanoscale level, when additionally to the stable graphene-like geometry, the two-dimensional square-octagon phase was predicted with surface orientation corresponding to the (001) plane of body-centered-tetragonal (bct) crystal [11,20,21,22,23,24]. On the other hand, the wurtzite phase is found to be metastable while it is more favorable in the bulk state [7]. The 2D bct-phase displays remarkable properties, e.g., exceptionally high carrier mobility [25] and it is intriguing why such a promising 2D crystal has not been observed in experiment yet. To answer that, a careful examination of the subtle thermodynamic balance of different 2D phases and a clear understanding of surfaces effects interplay are necessary. The latter requires to take into account surface functionalization with a necessary consideration of the possible “chemically induced phase transition” between the phases after the surface functionalization, e.g., by hydrogen.

In the current work we addressed these questions by careful investigation of the 2D AlN and GaN stability. We studied the features of film transition from bulk-like wurtzite phase to graphene-like g-AlN, g-GaN and further to 2D body-centered-tetragonal phase bct-AlN, bct-GaN. We predicted possible control of the atomic structure of 2D AlN and GaN by adsorption of hydrogen atoms to the surface and obtained that a chemically induced phase transition allows the production of bilayered films with uniform bct structures, whereas adsorption of H atoms to thicker films leads to an additional stabilization of this phase.

## 2. Methods

Multilayered AlN and GaN structures were studied using density functional theory (DFT) [26] as implemented in the Vienna ab initio simulation program package (VASP) [27,28,29].

The subject of this work, the relative stability of the phases, requires an accurate calculation of the potential energy of various configurations of the studied chemical compositions. Therefore, the DFT methods used have been repeatedly tested for agreement with experimental data, such as the phase transition pressure and crystal lattice parameters as well as compared with the results of theoretical articles using similar methods [30]. Thus, the projector-augmented wave (PAW) [31] method was chosen for quantum-mechanical calculations with the periodic boundary conditions (PBC). The Perdew–Burke–Ernzerhof generalized gradient approximation (GGA-PBE) [32] was adopted as the exchange-correlation functional. To ensure sufficient calculation accuracy the plane wave basis with the energy cutoff of 400 eV was used and the Brillouin zone sampling was made by the Monkhorst–Pack scheme [33] with the step of 0.03 × 2π/Å. Atomic structure optimization was carried out until the forces acting on each atom became smaller than 10^−2^ eV/Å.

Since many 2D crystals have a layered structure, it is necessary to take into account the van der Waals interaction between the layers. Therefore it was decided to include the vdW-DF correction [34] and accordingly, all tests were rechecked.

The slab simulation with PBC conditions requires careful choice of vacuum region in the perpendicular to the film’s plane direction. The energy of films was tested versus size of c-lattice vector. In order to avoid interaction between adjacent cells with periodic boundary conditions, the vacuum size of at least 15 Å was applied.

## 3. Results and Discussion

With decreasing structure size, the surface effects start to play a major role in phase stability. In the case of ultrathin films it can be expected that the thermodynamic potential (internal energy E in our case) is proportional to the ratio of surface area to volume, i.e., to the inverse film thickness or inverse number of layers, 1/n, with linear tendency to the bulk energy. By plotting of the dependencies in the corresponding axes relative stability of any 2D phases can be described.

**Figure 1:**
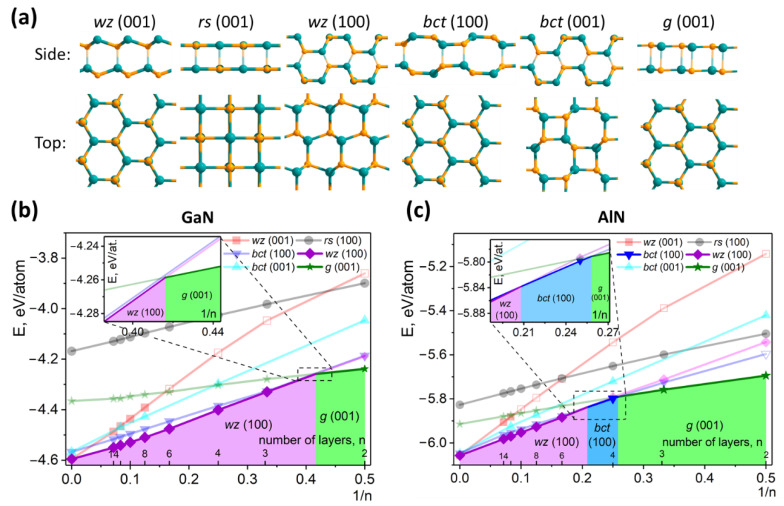
(**a**) Side and top views of 2D-GaN and 2D-AlN phases; (**b**,**c**) dependence of 2D-GaN and 2D-AlN films energy on the inverse number of layers, 1/n (horizontal axis also contains ticks denoting noninverted n). The region of phase transition is zoomed and presented in the inset. Pale colors depict the energy dependencies of less favorable phases.

We investigated the stability of films with number of layers varying from 2 to 14 (Figure 1) considering the most common phases for AlN and GaN compounds which could be stable in 2D case. Among them there are polar wurtzite ((wz (001)), nonpolar wurtzite (wz (100)), rocksalt (rs (100)) and some specific phases such as graphene-like (g (001)), body-centered-tetragonal bct (100) and bct (001) (which monolayered structure is called as haeckelite [20,21,22,23]). To avoid misunderstanding here we need to define a “layer” term. In Figure 1a the side view of each investigated structure represents two “layers” connected with transparent bonds. In this notation all films have AA’ stacking in direction perpendicular to the film plane. Thus, the cation-anion planes morphed into A or A’ layer are counted as one “layer”.

In the case of bilayered structures for both systems the graphene-like phase is the favorable one. Its structure possess the most stable stacking with the superposed hexagons of neighboring planes where Ga(Al) and N atoms alternate along the c direction in agreement with previous works [35,36]. The bilayered polar wurtzite film for both materials is the least energetically favorable one and the atomic structure is even unstable (due to divergence of the surface energy), illustrated by empty markers in the corresponding plot. For this reason, in our simulation relaxation of the low-dimensional wurtzite phase was carried out with a set of constraints. In particular, Ga(Al) and N atomic positions were frozen along the c direction that prevented the film splitting to the g-GaN or g-AlN. Such effect was found in various 2D compounds and called as “ionic graphitization” [5,6,7]. For the case of AlN three-layered film also has graphene-like structure.

With film thickness increasing other phases of GaN/AlN become energy favorable (see Figure 1b,c). Thus, from 2 (GaN) and 4 (AlN) layers special bct phase with the 8|4 bonding motif is more stable than graphene-like phase which was already predicted for the case of GaN [24,25]. We estimated that bct phase is energy favorable for the films up to the thickness of ~33 (GaN) and ~100 (AlN) layers after which wurtzite films with polar surface becomes energy preferable. Moreover, the energies of the bulk bct and wurtzite phases are still very close: difference between bulk wz-GaN and bct-GaN (wz-AlN and bct-AlN) is only 0.023 eV/atom (0.009 eV/atom). It raises a question why such phase was never experimentally observed both for GaN and AlN?

For the case of low-dimensional structures the lack of observation of bct phase can be explained by taking into account the energy of the wurtzite films with nonpolar (100) surface. These films display much lower surface energy due to the disappearance of the destabilized dipole moment induced by charge transfer in polar surface. wz-GaN (100) and wz-AlN (100) films become more energy favorable than g-GaN and g-AlN film for more than two and three layers, respectively, and overlap the energies of corresponding bct-GaN and bct-AlN (100) films. bct-AlN (100) and wz-AlN (100) films are equally stable for the number of layers between 4 and 6. Thicker films display the most stable wurtzite structure with a nonpolar surface too.

The appearance of bulk-bct is hindered not only by unfavorable energetics of nanoscale structures needed for further growth but by negative pressure of phase transition as well. Higher energy and lower density of bct-phase than corresponding wz-phase result in negative transformation pressure. These values are −5.5 GPa for GaN and −2.8 GPa for AlN as found from $-\frac{E_{\mathrm{wz}}–E_{\mathrm{bct}}}{V_{\mathrm{wz}}–V_{\mathrm{bct}}}$, where *E*_wz_, *V*_wz_ and *E*_bct_, *V*_bct_ are energy and unit volume of the wz and bct phases, respectively. This means that the pressure-induced conversion of wz to bct is unattainable with modern experimental methods. In contrast, energetically less favorable rocksalt phase possesses positive value of phase transition pressure and is observed experimentally at high pressures [37,38]. Additionally, the bulk bct phase is considered as an intermediate state between the rocksalt and wurtzite structures [30].

We can propose that the bct phase can exist only at the nanoscale level in the constraints of hexagonal symmetry. As seen from Figure 1a, three films with the structure of polar wurtzite, bct (100) and graphene-like have hexagonal symmetry with almost the same in-plane lattice vectors. Specific symmetry of these phases can distinguish them from more energy favorable nonpolar wurtzite. Thus, hexagonal substrate constraining the symmetry of the grown films [9,39] can make hexagonal films formation to be favorable whereas the symmetry mismatch between the substrate and nonpolar wurtzite (100) can overcome the thermodynamic advantage of this phase.

In addition to the issue of energy stability it is helpful to estimate the barriers of transformations from one phase to another. wz (001), bct (100) and g (001) films have almost the same in-plane lattice constant and therefore differ only by atomic coordinates in normal direction. So, the intermediate states of transformation from polar wurtzite through graphene-like to bct phase could be easily constructed by optimization of structures with constrained z-coordinate.

**Figure 2:**
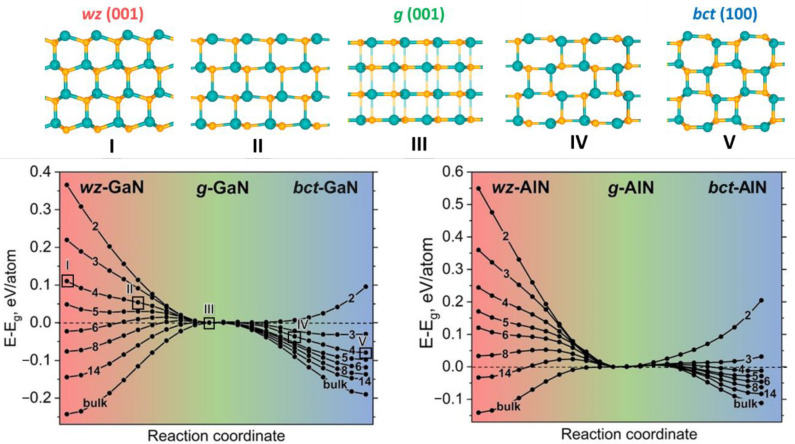
(**top**) The transition process from the wurtzite to the bct phase through graphene-like phase; (**bottom**) barriers of transformation of the GaN, AlN thin films from wz (001) to bct (100) structure. The energy of the graphene-like phase is taken as zero and marked by horizontal dashed line. The points corresponding to the top structures are marked by empty squares and the Roman numerals.

Considering the constraints of hexagonal symmetry we have studied the transition process from wz (001) to bct (100) film through graphene-like structure for GaN and AlN with different number of layers (Figure 2). To describe a possible atomic pathway, we gradually shifted the atoms in the z direction.

The specific case of bilayered film (and three-layered for the AlN) is clearly observed when graphene-like phase has energy minimum in comparison with wurtzite and bct phases. The further increase of the films thickness leads to the minimum reduction to inflection point and then to maximum point. In the case of 3–4 layers of GaN (as well as 5–6 layers for the AlN) only one energy minimum appears for the bct phase which is obtained by transformation from the wz-GaN (wz-AlN) without any barrier. For the case of GaN this result corresponds with the work of Kolobov et al. [24]. With a further increase of the layers number the transition energy barrier between wurtzite to bct phase appears.

The analysis of transformation barriers strengthens the criteria of relative stability of the three chosen phases. Whereas the thermodynamics shows that bct (100) film more favorable up to ~33 (GaN) and ~100 (AlN) layers, in Figure 2 we can see that phase transformation from wz (001) to bct (100) can spontaneously proceed only up to ~6 (GaN) and ~14 (AlN) layers. Moreover, the graphene-like phase thicker than 2 layers is not even locally stable and transforms into wz (001) or bct (100) without any barrier. It should be noted also that in the case of thick films or bulk GaN (AlN) the transformation barriers are ~0.1–0.2 eV which are unlikely overcome by thermal fluctuations (~0.03 eV at room temperature), while surface chemistry might lower the barriers and turn the balance from one phase to another [2]. It was shown for the chemically induced phase transition of graphene to diamane where the chemical binding with the catalyst atoms leads to change in the chemical activity of the graphene atoms and facilitates the chemical reaction [2,3,4]. The hydrogen atom adsorption onto multilayered graphene films surface induces the transformation of the chemically inert sp^2^-hybridized graphene lattice to the sp^3^-hybridized diamond structure which cannot be obtained in other ways. Therefore, it can be expected that relative stability of 2D phases of GaN and AlN can be changed by adsorption of chemical groups on the surface of films.

**Figure 3:**
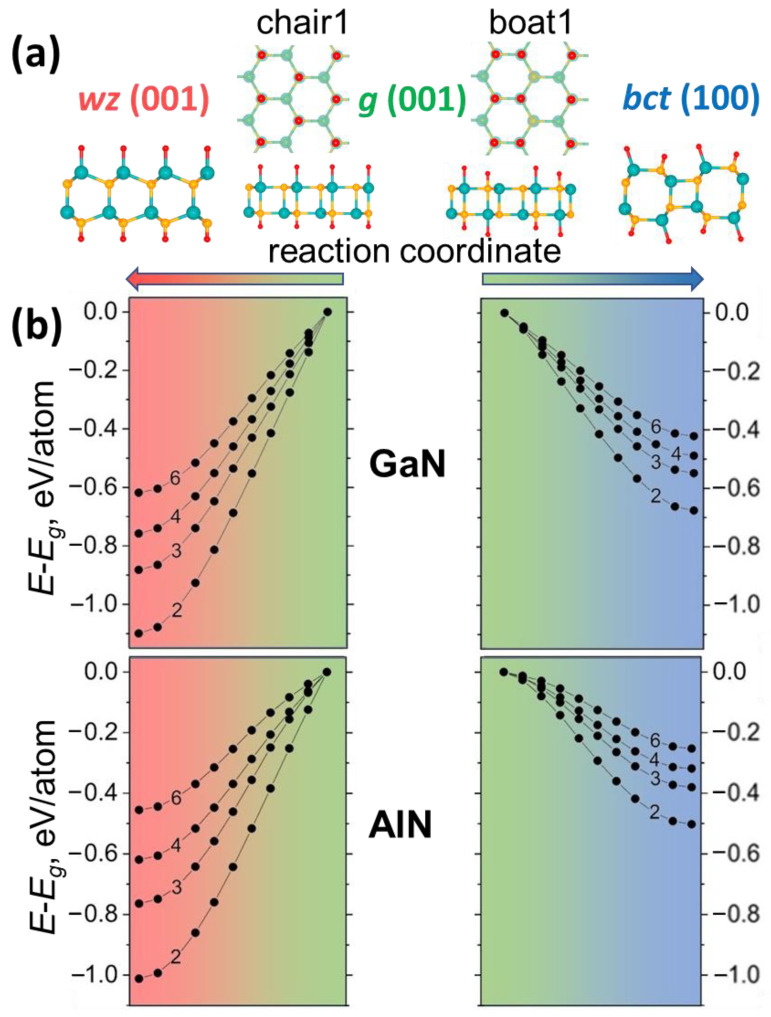
(**a**) Top and side view of GaN and AlN graphene-like films with hydrogen in position “chair1” and “boat1”. These films transform into wz (001) or bct (100) (side view is shown) depending on the arrangement of H atoms. (**b**) The transformation barriers of the g-GaN and g-AlN thin films with hydrogenated surface to the bct and wurtzite phase. The energy of the hydrogenated graphene-like film is taken as zero.

Graphene-like flat surface is open for any functionalization pattern and the organization of adatoms on the film surface is critical in this issue. Moreover, it may depend on adatom type and external conditions. Hydrogen atoms can be located on the film surface in various positions “chair1”, “boat1”, “boat2”, “chair2” and cause different transformations in case of different stackings of graphene-like phase. In the case of more energetically favorable stacking with Ga (Al) atoms above N there are two possible positions of hydrogen atoms on the surface which leads to the formation of a close-packed structure, “chair1” and “boat1” (Figure 3a).

As a result of a chemically induced phase transformation we observed the transition of the graphene-like phase to wurtzite in the case of “chair1” and graphene-like to bct in the case of “boat1” (Figure 3b). Even two layered hydrogenated graphene-like film becomes unstable in contrast to results of thermodynamic analysis of the films with clean surface. On the other hand, it is clearly seen that hydrogen atoms stabilize thin wz and bct films, as was expected by dangling bonds saturation.

Summing up, searching for the ways of new bct phase formation in GaN and AlN compounds requires to consider a set of criteria: the thermodynamics demonstrates the area of stability of bct phase as ultrathin films in the constraints of hexagonal symmetry, chemistry can just stabilize the film but not to facilitate the phase transition except special bilayered (and three-layered AlN) case. Thus, making the bct-films seems like is challenging but not impossible task.

## 4. Conclusions

In conclusion, in the current work stability of ultrathin films of GaN and AlN were investigated by means of first-principles calculations. In particular, we have studied the area of phase stability of GaN and AlN such as polar wurtzite (001), nonpolar wurtzite (100), graphene-like (001), body-centered-tetragonal bct (100), bct (001) and rocksalt (100) phases. We represented their energy profile as a function of film thickness and shown that for GaN systems graphene-like phase is stable only for the thinnest bilayer films whereas for AlN both two- and three-layered graphene-like films are favorable. With increase of the thickness, bct (100) film becomes more energy favorable than corresponding polar wurtzite and graphene-like films. However, wurtzite structure with nonpolar (100) surface overlaps the energies of bct-GaN (AlN).

Focusing on the question of stability of the less studied and potentially more interesting for experiment bct phase, we considered hexagonal symmetry confinement which possibly can prohibit the growth of the unwanted nonpolar wurtzite. Then, the process of transition from wurtzite to the bct phase has been investigated in detail. The graphene-like phase was taken as an intermediate state for thin films with different number of layers. It is shown that the films phase transformation from wz (001) to bct (100) can be spontaneously proceeded only up to ~6 (GaN) and ~14 (AlN) layers. Moreover, the graphene-like film thicker than 2 layers for GaN (more than 3 layers in the AlN case) is not even locally stable and transforms into wz (001) or bct (100) films without any barrier. Finally, studying the surface chemicals influence we recalculated the barriers of transformations of wz (001) to bct (100) films and have shown how hydrogen adsorption supports the stability of these phases depending on the H adatoms arrangement under external conditions.

## Data Availability

MDPI Research Data Policies.
